# Gene expression analysis reveals the tipping points during infant brain development for human and chimpanzee

**DOI:** 10.1186/s12864-020-6465-8

**Published:** 2020-03-05

**Authors:** Hui Tang, Ying Tang, Tao Zeng, Luonan Chen

**Affiliations:** 1 Key Laboratory of Systems Biology, CAS Center for Excellence in Molecular Cell Science, Shanghai Institute of Biochemistry and Cell Biology, Shanghai Institutes for Biological Sciences, Chinese Academy of Sciences, University of Chinese Academy of Sciences, Shanghai, 200031 China; 2 CAS Center for Excellence in Animal Evolution and Genetics, Chinese Academy of Sciences, Kunming, 650223 China; 3 Shanghai Research Center for Brain Science and Brain-Inspired Intelligence, Shanghai, 201210 China

**Keywords:** Brain development, Gene expression, Bioinformatics, Tipping point, Dynamic network biomarker

## Abstract

**Background:**

Postpartum developmental delay has been proposed as an important phenotype of human evolution which contributes to many human-specific features including the increase in brain size and the advanced human-specific cognitive traits. However, the biological processes and molecular functions underlying early brain development still remain poorly understood, especially in human and primates.

**Results:**

In this paper, we comparatively and extensively studied dorsolarteral prefrontal cortex expression data in human and chimpanzee to investigate the critical processes or biological events during early brain development at a molecular level. By using the dynamic network biomarker (DNB) model, we found that there are tipping points around 3 months and 1 month, which are crucial periods in infant human and chimpanzee brain development, respectively. In particular, we shown that the human postnatal development and the corresponding expression changes are delayed 3 times relative to chimpanzee, and we also revealed that many common biological processes are highly involved in those critical periods for both human and chimpanzee, e.g., physiological system development functions, nervous system development, organismal development and tissue morphology. These findings support that the maximal rates of brain growth will be in those two critical periods for respective human and primates. In addition, different from chimpanzee, our analytic results also showed that human can further develop a number of advanced behavior functions around this tipping point (around 3 months), such as the ability of learning and memory.

**Conclusion:**

This work not only provides biological insights into primate brain development at a molecular level but also opens a new way to study the criticality of nonlinear biological processes based on the observed omics data.

## Background

The primate brain development was traditionally studied by investigating the conserved biological processes and functions across mammals [1]. Genetic changes resulting in protein changes are probably too few to account for the great phenotype differences between humans and chimpanzees, prompting the hypothesis that changes in gene expression are likely to drive major phenotypic differences between humans and other primates [2]. Human and chimpanzee also show a number of significant differences in morphology and numerous cognitive traits during development tendency [3–7]. Some detailed comparisons related to gene expression of human and chimpanzee have identified human accelerated regions [8, 9] or conserved noncoding sequences [10]. The individual ontogenesis of human and chimpanzee have mainly been compared in terms of skeletal morphology but there are few studies conducted at a molecular level. Results from these comparisons demonstrate that some human features may indeed be explained by neoteny, e.g. small jaws [11]. In human brain growth, developmental retardation or neoteny has been identified and also the brain developmental changes have been delayed comparing to other primate species. Besides, there is a well-known evidence that human’s infant brain develops sharply in the first 3 months in terms of morphology. The infant structural growth rate changes approximately from 1%/d to 0.4%/d at the end of 3 months [12]. Generally, the brain-growth rate of infant chimpanzee is three times as much as that of human.

The transcriptome is dramatically remodeled during postnatal brain development [13]. However, the biological processes and biological functions during early brain development in human and primates have not yet been extensively studied so far. In particular, the brain development can be considered as a nonlinear biological process, which involves the gradual change and then drastic transition near the tipping point. Thus, detecting the tipping point and further revealing the related molecular functions as well as gene regulations are important to understand the brain development as a nonlinear dynamical process at a molecular level. In this paper, we investigate the critical processes or events during early brain development at a molecular level by extensively studying dorsolarteral prefrontal cortex expression data in human and chimpanzee. For the first time, we identified 3 months for human and 1 month for chimpanzee as their respective tipping points during their infant brain development, based on Dynamic Network Biomarker (DNB) theory [14, 15]. In addition, we found three times difference in terms of brain growth rate between human and chimpanzee due to human slow postnatal development. We also reveal many common biological processes involved in those key periods for both human and chimpanzee, e.g., physiological system development functions, nervous system development, organismal development and tissue morphology, etc. Actually, many published works have reported that the maximal rates of brain growth are 3 and 1 month for human and chimpanzee respectively, which is consistent with our analysis. Our analytic results also show that from around 3 months (tipping point) to later, human rather than chimpanzee can further develop a number of advanced behavior functions, e.g. the ability of learning and memory. This work not only provides biological insights into the brain development from a system viewpoint but also opens a new way to study the criticality of nonlinear biological processes based on DNB theory.

## Results

### Expression pattern analysis of dorsolateral prefrontal cortex expression data reveals smooth changes with drastic transitions during brain development

**Fig. 1:**
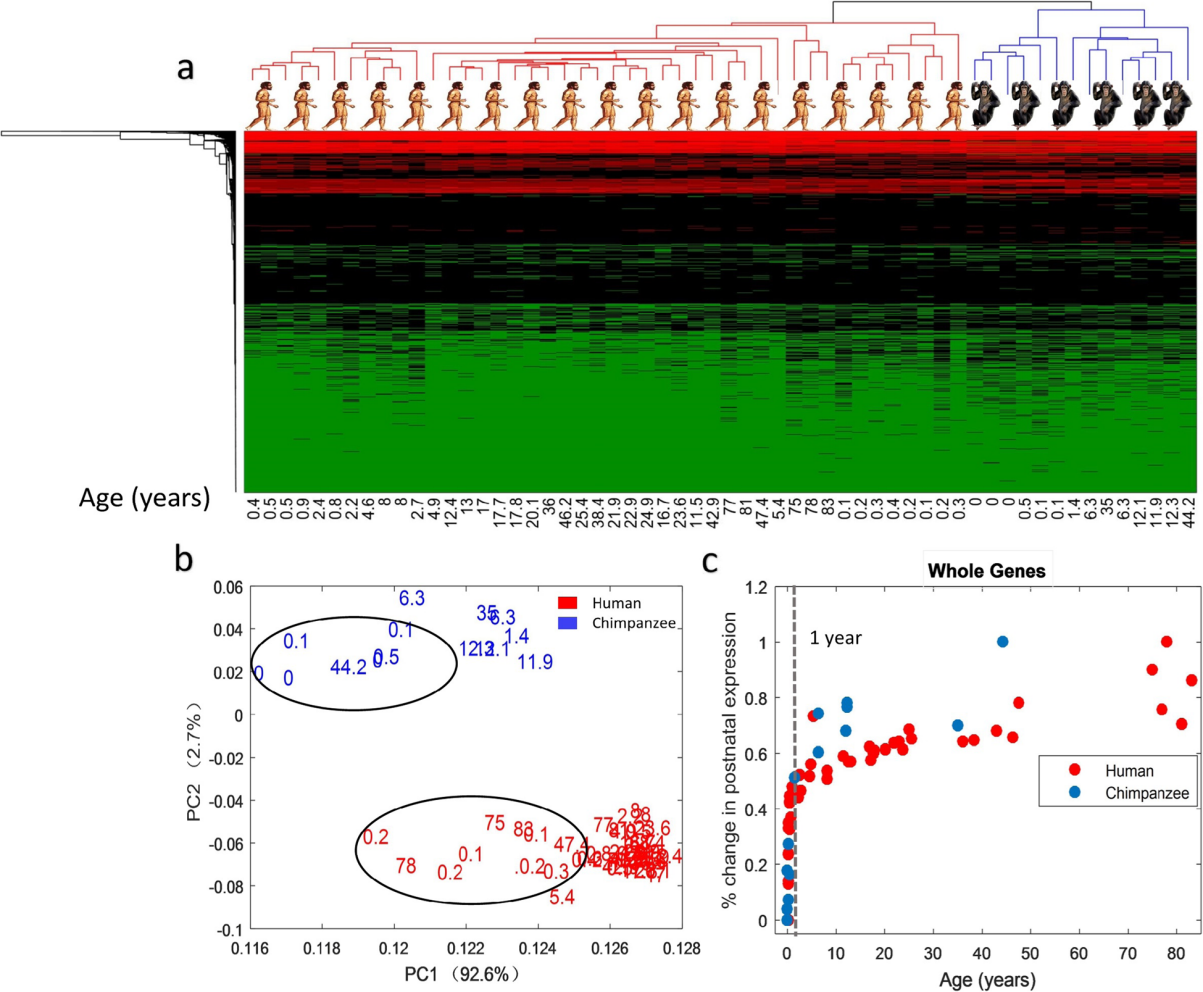
Gene expression analysis of dorsolateral prefrontal cortex expression data. **a** The clusters of the human and chimpanzee DLPFC dataset. **b** The first 2 principle components of the two species’ expression data by PCA. Each small dot represents the PC score along the top two principle components for each sample and the numbers represent each sample’s age in years. Blue represents chimpanzee samples and red represents human samples. For both humans and chimpanzees, the samples of the individuals’ ages (the most samples are included in circle) are distributed on early life stages. **c** Multi-dimensional scaling (MDS) plots show the first principal axes relative to the individual’s age in years. Each point represents a sample

We first evaluate the general expression pattern in the dorsolarteral prefrontal cortex (DLPFC) of three species, human, chimpanzee and macaque. We cluster these samples according to their expression levels by hierarchical clustering analysis. The samples are well separated into three clusters according to their species. Relative to macaque samples, chimpanzee is close to human in the resulting clusters in terms of the distance (Additional file 1). Meanwhile, human and chimpanzee also can be clustered into two different groups (Fig. 1a). Therefore, human and chimpanzee brains are considered very close but still different. Clearly, there are smooth changes with drastic transitions during the period. In this work, we focus on the analyses of infant brain development of human and chimpanzee, i.e. focusing on the early / first year life stages of humans and chimpanzees. In the principal component analysis (PCA) results of the data, we can also find that two groups, i.e. human and chimpanzee, are significantly separated (Fig. 1b). Next, we estimate when these gene expression changes take place during human and chimpanzee brain development and what the differences are between human and chimpanzee among the whole gene expressions. We used Multi-dimensional scaling (MDS) to evaluate the global changes of the whole samples in human and chimpanzee gene expressions relative to the individual’s age. We find that the most rapid changes or drastic transitions take place in the first year for both species. As shown in Fig. 1, the slope of the line for gene expressions is approximately 1 during the first year. More than 55% changes happened in the first year. Furthermore, the early trajectory of chimpanzee’s age-related gene expression changes is in close proximity to that of human (Fig. 1c).

### Dynamic network biomarkers identifies the tipping points during the infant brain development

**Fig. 2:**
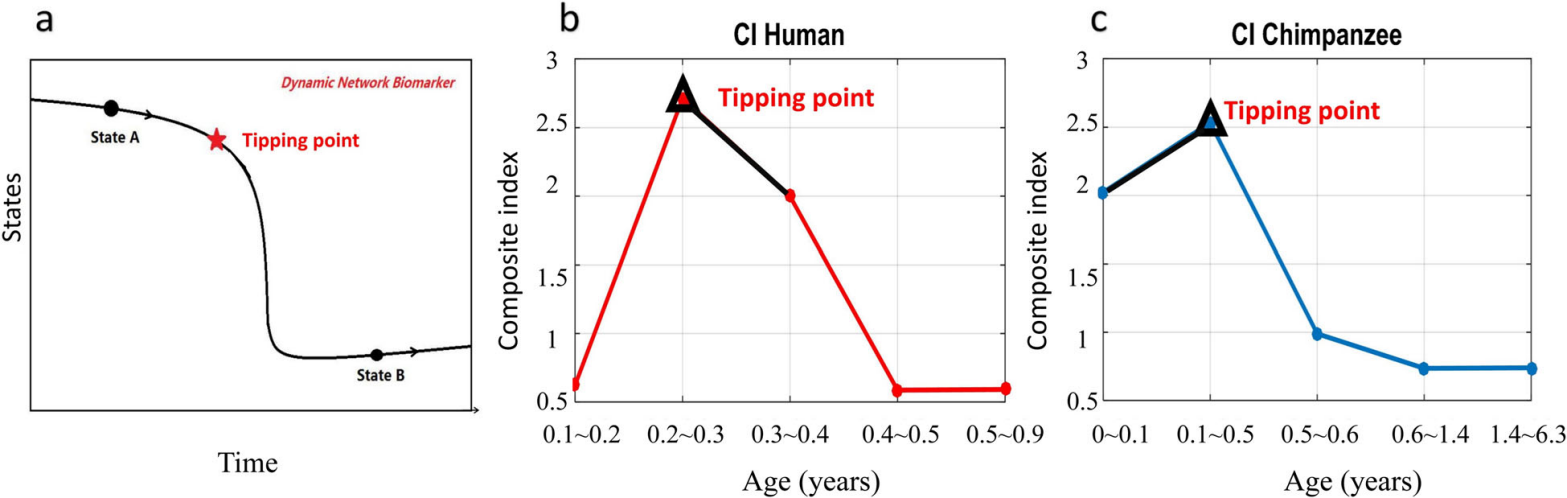
Detecting the tipping points for the infant brain development of human and chimpanzee. **a** Schematic illustration of the dynamical process from a state to another with a drastic transition for a biological system. **b** & **c** Detecting the tipping points during the infant brain development of two species by dynamic network biomarkers. **b** There are 13 infant human samples (age range from 0 to 0.9 years old). The two dots connected by the black line are significantly higher than other dots, and the corresponding time points involve 0.3. **c** There are 9 infant chimpanzee samples (age range from 0 to 6.3 years old). The two dots connected by the black line are significantly higher than other dots, and the corresponding time points involve 0.1. **b** & **c** represent the composite index of DNB (see Methods, and CI in Eq. (1))

Recent research works show that there is a sudden change or a critical transition during time evolution of many dynamic systems, such as climate system [16, 17], ecosystem [18, 19], economics, global finance [20, 21] and biological system [14]. Such a change plays a critical role in the development of whole system, and there will be a tipping point just before such drastic change or transition of system states. Owing to the sudden change, the dynamical features of biological system is from a state to another state through a drastic transition or transformation (Fig. 2a). To detect the tipping points during dynamic processes, a mathematical model, DNB theory with three quantitative criteria [14] was developed and has been widely applied to investigate many diseases [14, 15, 22–27] and biological processes [24, 28, 29] based on the observed data. In this work, DNB is firstly applied to find whether or not there is a tipping point in the primate brain growth in the first year (infant) and what biological functions are developed (enriched) before and after such tipping point.

**Fig. 3:**
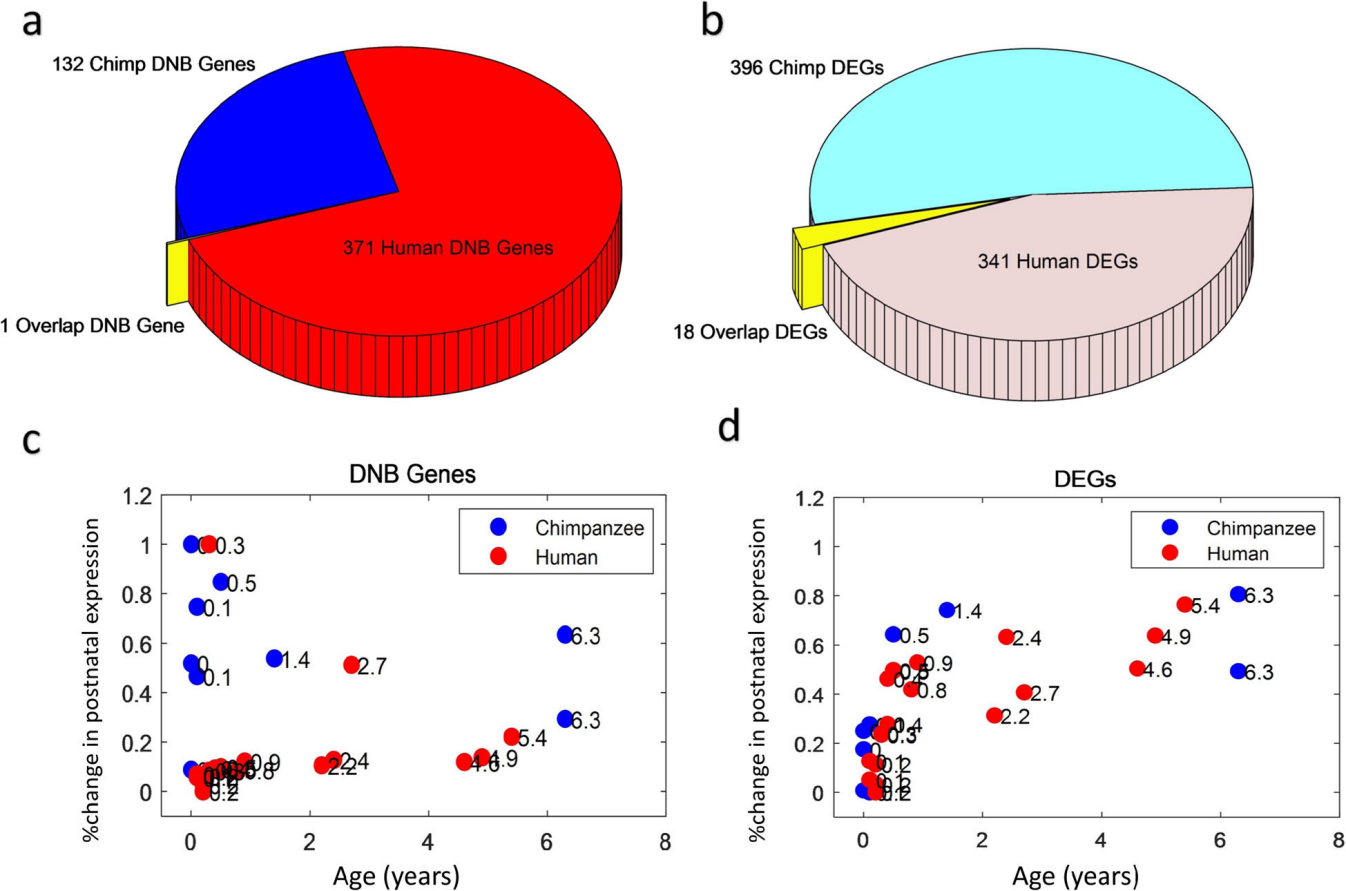
The global gene expression changes during brain growth. **a** & **b** The pie graphs of the DNBs and DEGs; **a** The DNBs of human and chimpanzee. The yellow is the overlap (1 gene) of two groups; **b** The DEGs of human and chimpanzee. The yellow is the overlap genes (18 genes) of two group DEGs; **c** MDS plot shows that the first principal axis relative to the individual’s age before 8 years old. Each point represents a sample, i.e., the sample’s global expression analysis by using DNBs (371 human genes and 132 chimpanzee genes); **d** The DEGs (341 human genes and 396 chimpanzee genes) are used to show the global expression tendency relative to age

To identify specific tipping points during the first year brain development of human and chimpanzee, the gene expression data include 13 infant humans and 9 infant chimpanzees were used. According to the three criteria of DNB for detecting tipping points [14], we analyzed all 17,429 genes. The composite index of the DNB suggests that human’s DLPFC gene expression has a significant change around 3 months (Fig. 2b) and around 1 month for chimpanzee (Fig. 2c). Additional file 2 also shows that 3 and 1 month are the tipping points of the infant human and chimpanzee respectively. By using the DNB model, we got 371 dynamical network biomarker genes (DNBs) (Fig. 3a, and Additional file 3) of human and 132 DNBs (Fig. 3a, and Additional file 3) of chimpanzee. There is one gene (TNFAIP3) overlapping the two specie-specific DNBs (Fig. 3a and Additional file 3). Meanwhile, we obtained 341 significantly differentially expressed genes (DEGs) around 3 months for human (Fig. 3b, and Additional file 4) (ANOVA false discovery rate (FDR) < 0.01 and fold-change > 2); and similarly, we detected 396 significant DEGs for chimpanzee (Fig. 3b, and Additional file 4). There are 18 genes overlapping the two DEGs (Fig. 3b and Additional file 4). Previous reports of these overlapping genes relevant to the functions of brain, especially on brain development, are listed in Table 1.

**Table 1:** Overlapping DNBs/DEGs between human and chimpanzee

	Type	Entrez gene name	Functions of brain development	Other functions in brain
RAET1E	DEG	retinoic acid early transcript 1E	[30]	
LEFTY2	DEG	left-right determination factor 2	[31]	
TUBB2B	DEG	tubulin beta 2B class IIb	[32]	
PPM1J	DEG	Protein phosphatase Mg2+/Mn2+ dependent 1 J	[33]	
CCDC182	DEG	coiled-coil domain containing 182		[34]
SIRPD	DEG	signal regulatory protein delta		[35]
OR5L2	DEG	olfactory receptor family 5 subfamily L member 2		[36]
FPR1	DEG	formyl peptide receptor 1		[37]
GPR34	DEG	G protein-coupled receptor 34		[38]
FCGRT	DEG	Fc fragment of IgG receptor and transporter		[39]
P2RY12	DEG	purinergic receptor P2Y12		[40]
AIF1	DEG	allograft inflammatory factor 1		[41]
SI	DEG	sucrase-isomaltase		
OR10H2	DEG	olfactory receptor family 10 subfamily H member 2		
CD14	DEG	CD14 molecule		
PIK3CG	DEG	phosphatidylinositol-4,5-bisphosphate 3-kinase catalytic subunit gamma		
RP11-1407O15.2	DEG	RP11-407P15.2 protein-coding		
TNFAIP3	DNB	TNF alpha induced protein 3		

According to the DNB theory, DNBs may have strongly functional impacts on biological processes at the tipping points. Next we examine whether or not there are significantly differential expression changes during the development of infant brain for DNBs. We find that the global expression tendency of DNBs is significantly stronger than other genes. Obviously, the relative growth rate of infant chimpanzee is much higher than human (Fig. 3c) especially in the first year. There is the similar tendency for the DEGs of the two groups (Fig. 3d). These results suggest that DNBs and DEGs may play important roles around the tipping points during the brain growth together. Therefore, by analyzing DNBs and DEGs, we have shown a rapidly brain development mechanism for the coordination of gene expression in infant chimpanzee relative to infant human.

### Advanced behavior related functions develop for human at the tipping point in contrast to chimpanzee

**Fig. 4:**
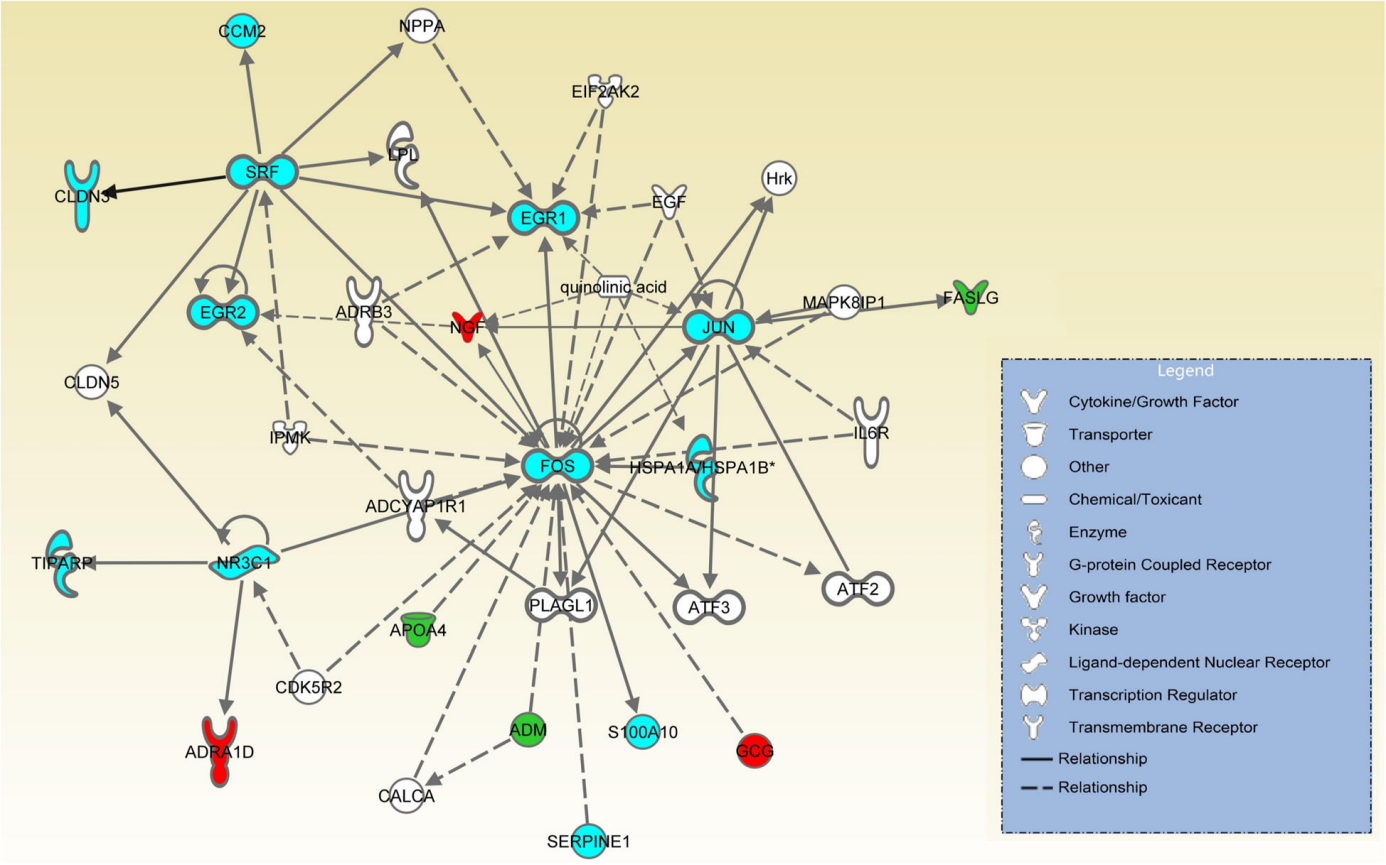
Top networks and pathways of human DEGs and DNBs. Blue genes are DNBs, red genes are the up-regulated genes along with time, and green genes are the down-regulated genes. DNBs play a leading role in this network

We carried out analysis of both human’s DNBs and DEGs before and after 3 months with IPA (Ingenuity Pathway Analysis) [42]. The most significant physiological system development and function terms from Disease and Function analysis are listed in Table 2. We also conducted a network analysis by IPA. In the top-ranked networks, these genes play an important role in Behavior (IPA disease and Function Term, *P*-value = 1.02E-16~1.39E-6), including memory (*P*-value = 1.39E-6), learning (*P*-value = 9.11E-9) and behavior (*P*-value = 1.02E-16) (Table 3). Besides, these genes are also abundant in the network related to Cell Morphology (IPA disease and Function Term, *P*-value = 5.42E-16~2.26E-03). For DNBs in this network (Fig. 4), i.e., FOS, JUN and EGR1 are regulated by many genes, while SRF and NR3C1 regulate other genes in the network. The FOS gene family consists of FOS, FOSB, FOSL1, and FOSL2. Leucine zipper proteins are encoded by FOS and can dimerize with proteins of the JUN family. FOS and JUN form the transcription factor complex AP-1. These proteins have been identified as regulators of cell proliferation, differentiation, and transformation. The protein encoded by EGR1 belongs to growth factor EGR family and functions as a transcriptional regulator. Its target genes play a role in differentiation and mutagenesis. Upstream analysis by IPA shows that FOS, JUN and EGR1 have the same upstream regulator, growth factor EGF and transcription regulator FOS. SRF encodes a ubiquitous nuclear protein and stimulates cell differentiation and proliferation. NR3C1 encodes glucocorticoid receptor and participates in inflammatory reactions, cell differentiation and proliferation in target tissues [43]. Based on the analyses above, clearly these DNBs are related to Behavior in the functional network, and they have strong co-function links with other genes.

**Table 2:** Top physiological system developments and functions enriched in human DNBs and DEGs

Name	*P*-value range	Molecules
Tissue Morphology	3.23E-02 - 5.77E-06	43
Cardiovascular System Development and Function	8.72E-04 - 8.72E-04	5
Nervous System Development and Function	4.27E-02 - 8.72E-04	39
Organ Morphology	3.23E-02 - 8.72E-04	22
Organismal Development	3.94E-02 - 1.04E-03	23

**Table 3:** Top relevant diseases and biological Functions of the human DEGs and DNBs network in Fig. 4

Diseases and Functions	*P*-value range	Molecules
Behavior	1.02E-16 - 1.39E-6	21
Behavior	1.02E-16	21
Learning	9.11E-9	10
Memory	1.39E-6	7
Cell Morphology	5.42E-16 - 2.26E-3	27
Morphology	5.42E-16 - 3.09E-7	25
Size	6.89E-11	12
hypertrophy	1.16E-10 - 2.26E-3	10
Sprouting	1.18E-10	11
Branching	1.32E-9 - 1.14E-6	10
depolarization	2.03E-7	5
Formation	4.52E-7	11
Neurogenesis	9.34E-7	9

Infants grow at the fastest rate at 3 months old, not only for their height and weight but also for their brain development. The head circumference will increase by about 1.25 cm during the third month. Their behavior undergoes huge changes during this period. The Moro reflex, which is developed in human evolution as a response to a sudden loss of support, starts disappearing gradually at this period [44]. When the soles of infant feet touch a flat surface, they will attempt to walk by placing one foot in front of the other: this phenomenon is called as walking reflex or stepping reflex. This behavior is present at birth and disappears around 10 weeks [45]. At 3 months old, infants’ unconscious activities, such as Moro reflex and Stepping reflect, disappears, meanwhile their behavior develops more purposefully and steadily towards maturation. In addition, during that period, hands begin to perform advanced functions and can open and close consciously. They start staring at something that interests them as well. Their Hand-eye coordination is also improving.

In a brief summary, our human DNBs and DEGs are found abundant in Nervous System Development and Function, Cell Morphology and Behavior. Nervous System Development and Cell Morphology support the fast brain development, so that, infants become to develop the advanced behavior. In the molecular level, we identify several DNBs as the hubs of the network, which play critical roles in regulating the relevant functional modules and pathways.

### Chimpanzee acquires the ability of basic behaviors associated with survival at the tipping point

By contrast, chimpanzee develops faster than human, which has been observed in many research works [13]. Actually, in this work we find that chimpanzee reaches the tipping point (1 month old) earlier. We also apply IPA analysis on chimpanzee DNBs and DEGs before and after 1 month old. The most significant physiological system development and function terms from disease and function analysis are listed in Table 4. Though chimpanzee DNBs and DEGs have few overlaps with human DNBs and DEGs, their top physiological system development and function terms are very similar, including nervous system development, organismal development and tissue morphology. We can conclude that chimpanzee experiences the basic brain development similar to human beings at the tipping point. We further investigate the ability of behaviors by the function analysis, i.e., the feeding (*P*-value = 3.92E-02). Human has more advanced behavior ability at the tipping point than chimpanzee, such as learning and memory, which are the footstone of our cognitive competence.

**Table 4:** Top physiological system developments and functions of chimpanzee DNBs and DEGs

Name	*P*-value range	Molecules
Embryonic Development	3.92E-02 - 1.98E-02	2
Nervous System Development and Function	3.92E-02 - 1.98E-02	3
Organismal Development	3.92E-02 - 1.98E-02	2
Tissue Morphology	1.98E-02 - 1.98E-02	1
Behavior	3.92E-02 - 3.92E-02	1

## Discussion and conclusion

Human beings have had dramatic enlargement in brain size during the evolution, and developed advanced cognitive ability. In this work, we aim to reveal major biological events happening to infant human brain in the development process. We applied the DNB theory to analyze the tipping points based on the dorsolateral prefrontal cortex expression data of both human and chimpanzee.

Generally, chimpanzee grows faster than human beings for the brain development; they reach the tipping point around 1 month old, much earlier than human. In contrast, human’s tipping point is around 3 months old, which has been reported as one of the golden age of brain development [12]. Then we carried out the differential gene expression analysis around the tipping points, and conducted function analysis on DEGs and DNBs with IPA. Chimpanzee and human have different DEGs and DNBs, but their DEGs and DNBs are related to same physiological system development functions, i.e., nervous system development, organismal development and tissue morphology. These functions support the fast development of brain at this time period. However, human further obtains more advanced behavior functions at the tipping point. Specifically, human infants have the ability of learning and memory while chimpanzee infants do not. In the molecular level, we identified several DNBs in the hubs of human top ranked network, which play a critical role in regulating the modules and having the functional impacts on the brain growth. They are of great importance due to the related functions with cellular proliferation and differentiation in common. This work not only provides biological insights into the brain development at a molecular level but also opens a new way to study the criticality of nonlinear biological processes based on the observed omics data. As a future topic, in contrast to the traditional correlation analysis, we will adopt the direct associations [12] between molecules to study the molecular mechanism of infant brain development at the network level.

## Methods

### Data set preparation

All dorsolarteral prefrontal cortex (DLPFC) expression data sets from the microarray experiments were downloaded from the National Center for Biotechnology Information Gene Expression Omnibus (GEO) with the accession numbers GSE11512 (GC HG-U133 Plus2.0 experiments). The data sets contain 44 human samples (ranging in age 0–80 years), 14 chimpanzee samples (ranging in age 0–44 years), and 9 macaque samples. In each sampling period, there are 1 to 5 samples for gene expressions (Additional file 5).

### Clustering analyses of data sets

Before analyzing the two data sets, we transformed the original data by using normalization method. In this way, the data of each sample is in a uniform distribution which suits better for our statistical analysis. One method of cluster algorithm is ‘clustergram()’ in MATLAB Library, and the results are shown in Fig. 1a. Another one is PCA, and the results are given in Fig. 1b. MDS is used to calculate a 1D summary measure of global expression relative to the individual’s age (Fig. 1c). The ‘pdist()’ function in MATLAB Library is used to evaluate the distance between two samples, and the ‘mdscale()’ in MATLAB Library is adopted to estimate the global expression of samples.

### Dynamic network biomarkers (DNB) analysis

Based on the nonlinear dynamical theory, a system is near the critical state if there is a dominant group of molecules, i.e. DNB. According to the DNB theoretical analysis [14], we proved that the following generic properties hold when the infant brain biological system reaches a critical time point.

- There exists a group of molecules of human or chimpanzee, whose average Pearson’s correlation coefficients (PCCs) of molecules drastically increase in absolute value.
- The average PCCs of molecules between this group and any others (i.e., between molecules inside this group and any other molecules outside this group) drastically decrease in absolute value.
- The average standard deviations (SDs) of molecules in this group drastically increase.

If all of these three conditions are satisfied simultaneously, we call this group a dominant group of the system, which will play an important role in early brain development.

Therefore, when a biological system approaches the tipping point; a dominant group of genes appear among all genes. With the gene expression data of all samples in one period, this dominant group can be quantified by the following composite index (DNB model):

1 $$ CI=: \frac{S{D}_d\bullet PC{C}_d}{PC{C}_o} $$

where *PCC*_*d*_ is the average Pearson’s correlation coefficient (PCC) between the genes in the dominant group of the same time period in absolute value; *PCC*_*o*_ is the average PCC between the dominant group and others of the same time period in absolute value; *SD*_*d*_ is the average standard deviations (SD) of the genes in the dominant group. These three criteria together construct the composite index (CI) [14, 15, 24–28, 46–51]. The CI is expected to reach the peak or increase sharply during the measured periods when the system approaches the tipping point, thus indicating the imminent transition.

We applied this DNB method to detect the tipping points during the infant human and chimpanzee brain development. In each sampling period of infant human and chimpanzee, there are 1–5 samples with gene expression profiles. In order to increase the reliability of DNB result, the slide window method is incorporated into DNB model to process data. We calculate these three criteria of human and chimpanzee (Additional file 2). In addition, the Matlab package of DNB and the operation methods of DNB model are available at http://sysbio.sibcb.ac.cn/cb/chenlab/software.htm

### Functional analysis

DNBs and DEGs for the two species are used for pathway enrichment analysis by IPA [42] (Additional file 6), and network analysis is used to investigate the correlations of DNBs and DEGs in IPA network analysis. All additional results are described in Additional file 6 in this paper.

## Supplementary information

**Additional file 1.** Hierarchical cluster analysis of normalized DPLEC datasets (Human, Chimpanzee, Monkey), based on 17,429 expressed genes. The red represents human, the blue represents chimpanzee and the yellow represents macaque.

**Additional file 2.** Detecting the tipping points for the infant brain development of human and chimpanzee. Detecting the tipping points for two data sets, human (a, b, c, d) and chimpanzee (e, f, g, h). The infant human contain 13 samples (age range from 0 to 0.9 years old). The infant chimpanzee contains 9 samples (age range from 0 to 6.3 years old). Subfigures a and e represent the composite index (see Methods, CI in Eq.(1)), Subfigures b and f represent the mean SDs in the DNB of human and chimpanzee (see Methods, SD in Eq.(1)), Subfigures c and g represent PCCs in the DNB (see Methods, PCCd in Eq.(1)), Subfigures d and h are the PCCs between the DNB and other molecules (see Methods, PCCo in Eq.(1)).The results of the figure show the effectiveness of the DNB model by using our data sets and 3 and 1 month are the tipping points of two species.

**Additional file 3.** 371 DNBs of human and 132 DNBs of chimpanzee. There are one gene which are overlaps of two parts DNBs.

**Additional file 4.** The DEGs of human and chimpanzee around the tipping point. The overlap genes between DNBs and DEGs of two species.

**Additional file 5.** Sample Characteristics.

**Additional file 6.** Detailed DNB genes and DEGs IPA analysis summary and network analysis results.

## Data Availability

All data generated or analysed during this study are included in this published article and its supplementary information files.

## Abbreviations

DEG: Differentially expressed gene
DEGs: Differentially expressed genes
DLPFC: Dorsolarteral prefrontal cortex
DNB: Dynamic network biomarker
DNBs: Dynamic network biomarker genes
MDS: Multi-dimensional scaling
PCA: Principal component analysis
PCC: Pearson’s correlation coefficient
SD: Standard deviations
