# Supplementary figures and images for: Gene expression analysis reveals the tipping points during infant brain development for human and chimpanzee
Source: BMC Genomics. 2020 Mar 5;21(Suppl 1):74. doi: 10.1186/s12864-020-6465-8 (PMC7057467; doi:10.1186/s12864-020-6465-8)

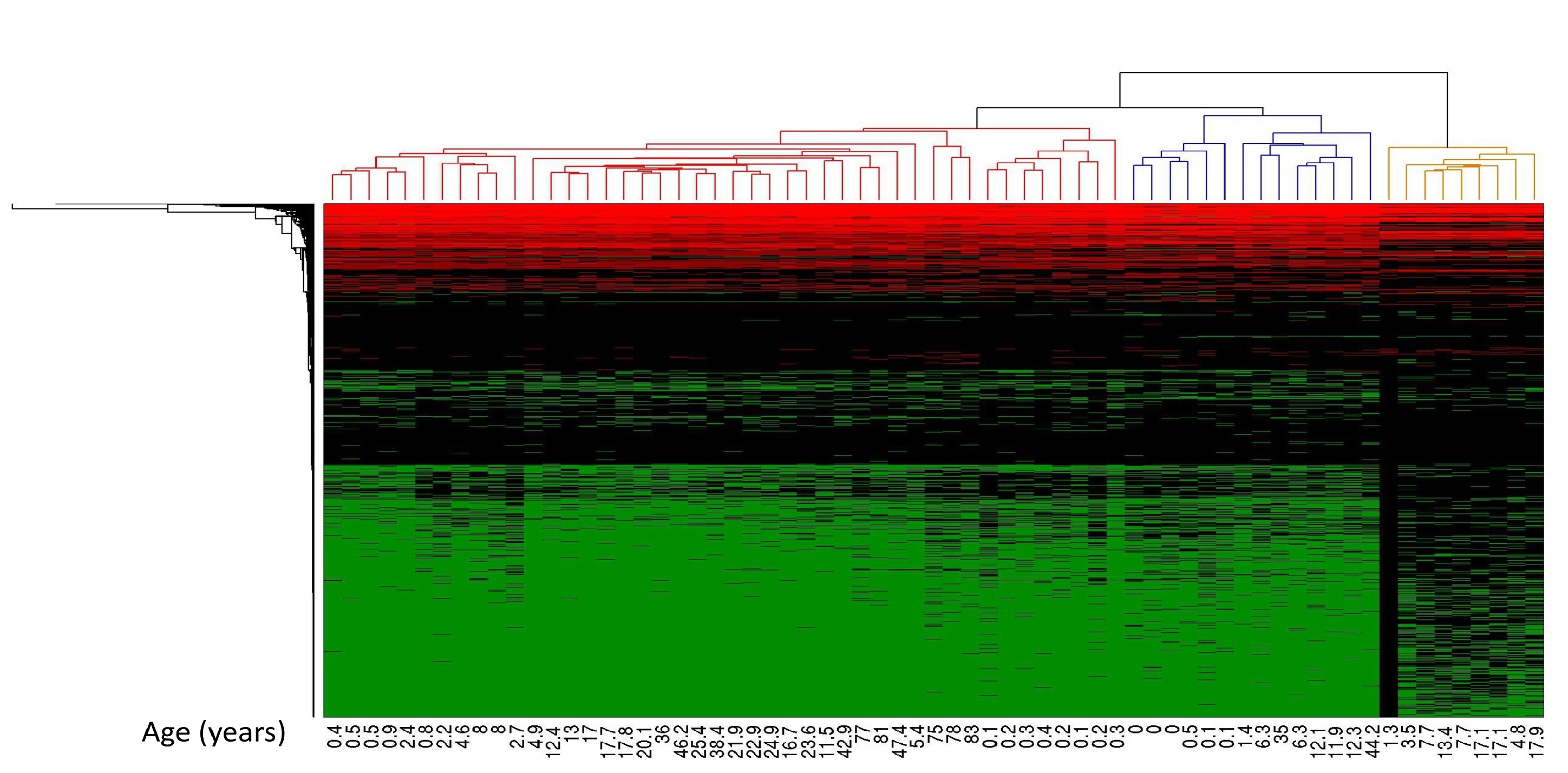

Supplement: Supplementary file 1 — Additional file 1. Hierarchical cluster analysis of normalized DPLEC datasets (Human, Chimpanzee, Monkey), based on 17,429 expressed genes. The red represents human, the blue represents chimpanzee and the yellow represents macaque. [file 12864_2020_6465_MOESM1_ESM.jpg]

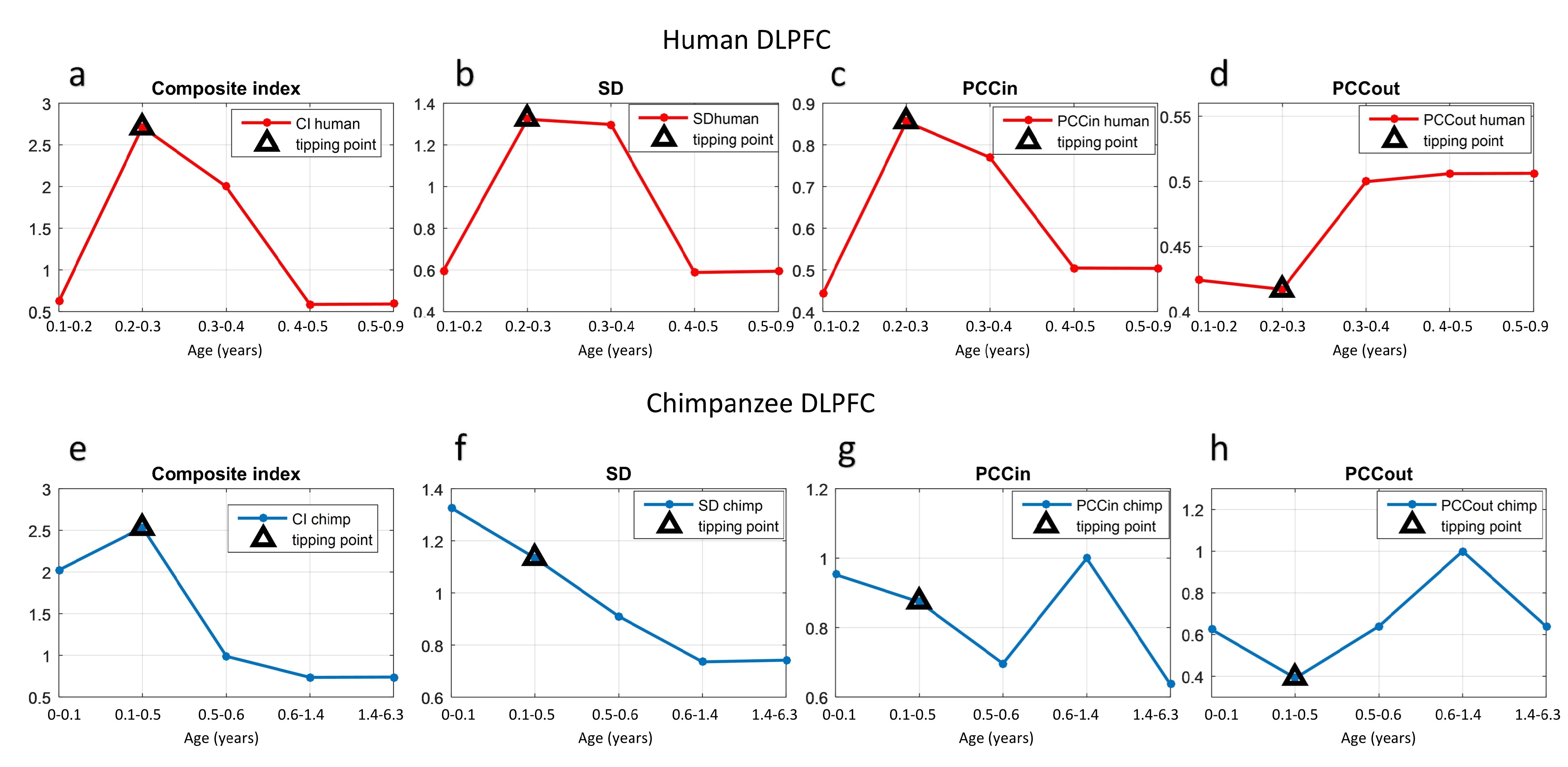

Supplement: Supplementary file 2 — Additional file 2. Detecting the tipping points for the infant brain development of human and chimpanzee. Detecting the tipping points for two data sets, human (a, b, c, d) and chimpanzee (e, f, g, h). The infant human contain 13 samples (age range from 0 to 0.9 years old). The infant chimpanzee contains 9 samples (age range from 0 to 6.3 years old). Subfigures a and e represent the composite index (see Methods, CI in Eq.(1)), Subfigures b and f represent the mean SDs in the DNB of human and chimpanzee (see Methods, SD in Eq.(1)), Subfigures c and g represent PCCs in the DNB (see Methods, PCCd in Eq.(1)), Subfigures d and h are the PCCs between the DNB and other molecules (see Methods, PCCo in Eq.(1)).The results of the figure show the effectiveness of the DNB model by using our data sets and 3 and 1 month are the tipping points of two species. [file 12864_2020_6465_MOESM2_ESM.jpg]
