# Supplementary material for: Gene expression analysis reveals the tipping points during infant brain development for human and chimpanzee
Source: BMC Genomics. 2020 Mar 5;21(Suppl 1):74. doi: 10.1186/s12864-020-6465-8 (PMC7057467; doi:10.1186/s12864-020-6465-8)
Supplement: Supplementary file 5 — Additional file 5. Sample Characteristics. [file 12864_2020_6465_MOESM5_ESM.doc]

Additional file Sample Characteristics.

|  | Species | Brain Region | Age Days | Age Years |
| --- | --- | --- | --- | --- |
| 1 | *Homo sapiens* | DLPFC | 39 | 0.1 |
| 2 | *Homo sapiens* | DLPFC | 54 | 0.1 |
| 3 | *Homo sapiens* | DLPFC | 56 | 0.2 |
| 4 | *Homo sapiens* | DLPFC | 62 | 0.2 |
| 5 | *Homo sapiens* | DLPFC | 89 | 0.2 |
| 6 | *Homo sapiens* | DLPFC | 92 | 0.3 |
| 7 | *Homo sapiens* | DLPFC | 118 | 0.3 |
| 8 | *Homo sapiens* | DLPFC | 139 | 0.4 |
| 9 | *Homo sapiens* | DLPFC | 141 | 0.4 |
| 10 | *Homo sapiens* | DLPFC | 188 | 0.5 |
| 11 | *Homo sapiens* | DLPFC | 198 | 0.5 |
| 12 | *Homo sapiens* | DLPFC | 301 | 0.8 |
| 13 | *Homo sapiens* | DLPFC | 332 | 0.9 |
| 14 | *Homo sapiens* | DLPFC | 801 | 2 |
| 15 | *Homo sapiens* | DLPFC | 893 | 2 |
| 16 | *Homo sapiens* | DLPFC | 990 | 2 |
| 17 | *Homo sapiens* | DLPFC | 1692 | 4 |
| 18 | *Homo sapiens* | DLPFC | 1773 | 4 |
| 19 | *Homo sapiens* | DLPFC | 1969 | 5 |
| 20 | *Homo sapiens* | DLPFC | 2920 | 8 |
| 21 | *Homo sapiens* | DLPFC | 2920 | 8 |
| 22 | *Homo sapiens* | DLPFC | 4213 | 11.5 |
| 23 | *Homo sapiens* | DLPFC | 4534 | 12 |
| 24 | *Homo sapiens* | DLPFC | 4733 | 12 |
| 25 | *Homo sapiens* | DLPFC | 6090 | 16.7 |
| 26 | *Homo sapiens* | DLPFC | 6222 | 17 |
| 27 | *Homo sapiens* | DLPFC | 6456 | 17 |
| 28 | *Homo sapiens* | DLPFC | 6505 | 17 |
| 29 | *Homo sapiens* | DLPFC | 7350 | 20.1 |
| 30 | *Homo sapiens* | DLPFC | 8006 | 21.9 |
| 31 | *Homo sapiens* | DLPFC | 8364 | 22.9 |
| 32 | *Homo sapiens* | DLPFC | 8263 | 23.6 |
| 33 | *Homo sapiens* | DLPFC | 9098 | 24.9 |
| 34 | *Homo sapiens* | DLPFC | 9262 | 25.4 |
| 35 | *Homo sapiens* | DLPFC | 13138 | 36 |
| 36 | *Homo sapiens* | DLPFC | 14024 | 38.4 |
| 37 | *Homo sapiens* | DLPFC | 15672 | 42.9 |
| 38 | *Homo sapiens* | DLPFC | 16855 | 46.2 |
| 39 | *Homo sapiens* | DLPFC | 17317 | 47.4 |
| 40 | *Homo sapiens* | DLPFC | 27375 | 75 |
| 41 | *Homo sapiens* | DLPFC | 28105 | 77 |
| 42 | *Homo sapiens* | DLPFC | 28470 | 78 |
| 43 | *Homo sapiens* | DLPFC | 29565 | 81 |
| 44 | *Homo sapiens* | DLPFC | 30295 | 88 |
| 45 | *Pan troglodytes* | DLPFC | 0 | 0 |
| 46 | *Pan troglodytes* | DLPFC | 1 | 0 |
| 47 | *Pan troglodytes* | DLPFC | 8 | 0 |
| 48 | *Pan troglodytes* | DLPFC | 40 | 0.1 |
| 49 | *Pan troglodytes* | DLPFC | 45 | 0.1 |
| 50 | *Pan troglodytes* | DLPFC | 186 | 0.5 |
| 51 | *Pan troglodytes* | DLPFC | 525 | 1.4 |
| 52 | *Pan troglodytes* | DLPFC | 2313 | 6.3 |
| 53 | *Pan troglodytes* | DLPFC | 2447 | 6.3 |
| 54 | *Pan troglodytes* | DLPFC | 4361 | 11.9 |
| 55 | *Pan troglodytes* | DLPFC | 4415 | 12.1 |
| 56 | *Pan troglodytes* | DLPFC | 4480 | 12.3 |
| 57 | *Pan troglodytes* | DLPFC | 12784 | 35 |
| 58 | *Pan troglodytes* | DLPFC | 16131 | 44 |
| 59 | *macaque* | DLPFC | 467 | 1.3 |
| 60 | *macaque* | DLPFC | 1269 | 3.5 |
| 61 | *macaque* | DLPFC | 1760 | 4.8 |
| 62 | *macaque* | DLPFC | 2814 | 7.7 |
| 63 | *macaque* | DLPFC | 2816 | 7.7 |
| 64 | *macaque* | DLPFC | 4894 | 13.4 |
| 65 | *macaque* | DLPFC | 6230 | 17.1 |
| 66 | *macaque* | DLPFC | 6230 | 17.1 |
| 67 | *macaque* | DLPFC | 6524 | 17.9 |

Abbreviations: DLPFC: Dorsolateral prefrontal cortex;
